# Loneliness Among Stroke Survivors with Mild Cognitive Impairment and Their Family Caregivers: A Qualitative Study

**DOI:** 10.1093/geroni/igaf122.1909

**Published:** 2025-12-31

**Authors:** Jiahui Xu, Ruotong Chu, Jing Wang, Juan Li, Bei Wu

**Affiliations:** Huashan Hospital Fudan University, Shanghai, Shanghai, China (People’s Republic); Huashan Hospital Fudan University, Shanghai, Shanghai, China (People’s Republic); University of New Hampshire, Durham, New Hampshire, United States; Huashan Hospital Fudan University, Shanghai, Shanghai, China (People’s Republic); NYU Shanghai, Shanghai, China (People’s Republic)

## Abstract

Loneliness is a significant challenge for stroke survivors with mild cognitive impairment (MCI) and their family caregivers, yet little is known about their unique experiences within social networks. This study explores how stroke survivors with MCI navigate loneliness, their psychological needs, and the strategies they use to cope. A descriptive qualitative study was conducted using semi-structured, in-depth interviews with 20 purposively selected stroke survivors with MCI and their family caregivers. All interviews were recorded, transcribed, and analyzed using thematic analysis. Five key themes emerged: (1) Enduring loneliness despite family presence. Survivors felt isolated even when surrounded by family due to a lack of meaningful interaction; (2) Concealing illness due to stigma. Fear of being judged or burdening others led many to hide their condition, further deepening isolation; (3) Limited awareness and neglect of cognitive impairment. Both survivors and caregivers often overlooked or misunderstood MCI symptoms, delaying support seeking; (4) Emotional vulnerability and defensive behaviors. Survivors struggled with feelings of shame, leading to withdrawal or defensive coping mechanisms; (5) Barriers to rehabilitation and social reintegration. A lack of personalized rehabilitation guidance and accessible social opportunities hindered survivors from rebuilding their confidence and social connections. The emotional barriers and social withdrawal experienced by both survivors and caregivers contribute to a cycle of isolation. Understanding these self-perceptions and the changing dynamics of social engagement is crucial for caregivers and healthcare providers to design interventions that foster meaningful connections and mitigate loneliness in this vulnerable group.

